# Remoteness decreases negative feelings about killing

**DOI:** 10.1186/s41235-026-00706-0

**Published:** 2026-02-24

**Authors:** Gary S. Katz, Ryan M. McManus, Rebecca B. Esquenazi, Andrew T. Ainsworth, Aaron Farnsworth, Abraham M. Rutchick

**Affiliations:** 1 California State University, Northridge, Los Angeles, USA; 2 Boston College, Boston, USA; 3 University of Washington, Seattle, USA; 4 United States Military Academy, West Point, USA

**Keywords:** Technology, Remoteness, Distance, Killing, Emotion, PTSD

## Abstract

Advancements in technology now afford surveillance and projection of force beyond the immediate vicinity. When people communicate or act via technology, feelings of subjective distance may attenuate affective and behavioral responses that would otherwise be experienced more intensely. As the psychological effects of technology on remoteness (and its consequent behavior) are thus far understudied, we had participants (N = 205) complete an ostensible insect-killing task in which participants were either close (in the same room) or remote (via webcam) from their targets. Participants who killed insects in the same room felt less negative emotional consequences than participants who killed remotely, suggesting that remoteness can blunt the immediate emotional consequences of killing. In addition, there was an indirect effect, such that participants in the remote condition felt less negative emotion and in turn killed more insects. Examining the features of remoteness that could potentially explain this effect, mediational analyses suggested that the subjectively experienced sense of distance from “killed” targets is the most promising candidate. Undoubtedly, remoteness-inducing technology can afford material benefits, such as the use of drones to decrease the number of soldiers on the ground. This research shows that the distance induced by remoteness can also reduce the immediate and potentially the longer-term emotional impact of killing. In combat and other military settings, as well as in civilian life, it is crucial to understand the influence of technological mediation and the distance it affords.

**Supplementary information:**

The online version contains supplementary material available at 10.1186/s41235-026-00706-0.

## Introduction

When people are remote from something they are judging, thinking about, or acting toward, they think and act differently. For example, when making a decision that would take effect in the distant future (versus immediately), people are more likely to endorse consequentialist behavior, such as killing one to save many (Aguilar et al., 2013). Related to this concept, transgressions viewed from a socially distant, third-party perspective are judged more harshly than those viewed from a first-person perspective (Eyal et al., 2008). Similarly, people more likely attribute others’ behavior to dispositions rather than situational factors when the behavior was spatially distant as opposed to spatially near (Henderson et al., 2006). Remoteness can stem from various sources: physical or temporal distance, lack of personal relevance (Boucher & Scoboria, 2015), immersion in social roles (Johnson & Downing, 1979; Peña et al., 2009), or through technological mediation. The current research investigates technologically mediated remoteness in the context of killing, experimentally manipulating remoteness in an insect-killing paradigm and assessing its affective and behavioral impact.

There is no doubt that killing is psychologically difficult (Jensen & Simpson, 2014). Direct exposure to killing or the result of killing is a primary criterion for post-traumatic stress disorder (American Psychiatric Association, 2022). Current diagnostic nomenclature excludes remote exposure (e.g., via electronic media or television) because remoteness may insulate people from the trauma. Consistent with the premise that proximity to killing increases the risk of trauma, soldiers who have killed are more likely to experience PTSD than those who only witnessed killing (Green et al., 1990; Van Winkle & Safer, 2011). This idea is further supported by evidence that sub-clinical symptoms of post-traumatic stress are lower in drone operators than in combat soldiers (Chappelle et al., 2014; but cf. Otto & Webber, 2013). To overcome the psychological difficulty of killing, military training often involves creating feelings of remoteness from the act (Grossman, 2009) and even dehumanization of the people who are killed (Browning, 1993; Smith, 2011).

In previous research, Rutchick et al. (2017) demonstrated that people killed more insects when remote from their targets (in another room) than when close to their targets (in the same room). In addition, participants remote from their targets who killed more insects felt less negative emotion when killing. The means by which remoteness exerted these effects is unclear, however, and one goal of the current study was to examine features of the remote context that could explain its impact on killing and emotion.

First, based on Construal Level Theory (Trope & Liberman, 2010), remoteness might induce a more abstract construal of killing. Abstract construals focus on broad purposes and higher-order goals, and in the context of killing these purposes are often framed positively: seeking justice, self-defense, and defending one’s country, for example (French & Jack, 2015). Conversely, the concrete details of killing are rarely framed positively, and there is evidence that abstract construals of drone strikes induce less concern with casualties (Powers, 2015). A second possibility is that being remote from the act of killing might lead people to place more importance on its benefits. In the current study, the task was framed as being scientifically and commercially valuable, and it may be that remoteness leads people to value those benefits more. This would also follow from Construal Level Theory, as abstract construals lead people to place more importance on broad moral principles (Eyal et al., 2008).

Third, being remote from the target may make the experience feel less vivid. Feeling disconnected from the act is a common response in soldiers who kill in combat (Maguen et al., 2009), and might serve to blunt the intensity of the emotions they experienced. Although the insect-killing task used in the current study clearly does not evoke the same intense emotions brought about by combat, an analogous process could unfold here, akin to the blunting process observed in previous work using insect-killing (Gibney et al., 2013). Last, it could be that subjectively perceived distance—that is, distance itself as experienced by participants—could change the nature of the experience. Although construal level and subjective distance have many similar effects, their affective consequences sometimes diverge (Moran & Eyal, 2022). There is evidence that, whereas abstract construals induce more positive thoughts and evaluations (Eyal et al., 2004), distance attenuates the intensity of felt emotion (Abraham et al., 2023; Williams et al., 2014). Thus, the subjective feeling of distance could drive the effect of remoteness.

The current study had two primary objectives: to replicate previous research examining the effects of remoteness on emotion and behavior, and to extend that research by examining potential features of remoteness that might impact these outcomes. As in previous work, participants used a remote-controlled machine to (ostensibly) kill ladybugs. Remoteness was manipulated: for some participants, the machine was in the same room, whereas for others the machine was in a different room. We hypothesized that participants who killed insects in the same room would kill fewer insects than participants in a different room, consistent with prior research. We also predicted that participants who killed insects in the same room would feel more negative emotion than those who killed remotely. With respect to the potential relevant contextual features, we had no *a priori* hypotheses; as described above, several candidates were explored.

## Method

### Participant recruitment and assignment to conditions

Undergraduate participants (initial *N* = 219) were recruited to the laboratory and were compensated with partial fulfillment of a course requirement. This sample size was chosen to yield 200 participants with usable data (which would yield a power of .80 to detect a between-subjects effect of Cohen’s *d* = .40, per GPower; Faul et al., 2007), oversampling to account for potential exclusions.

At recruitment, participants were told that the laboratory studied human factors psychology and that the study entailed testing a prototype apparatus. Upon reaching the laboratory, they were given more detailed instructions and informed that the study would involve using a machine to kill insects—specifically, ladybugs (*Hippodamia convergens)*. The cover story was elaborated to suggest the utility of an insect-killing machine (producing dye; extracting DNA for biological samples); this cover story was consistent with the department’s historical emphasis on human factors. At this time, two participants declined to participate. Participants were then shown how to use the machine; here, two more participants ended their participation. This yielded a sample of 215 participants (73% female, *M*_*age*_ = 19.31) who began the insect-killing task.

### Insect-killing procedure

The insect-killing paradigm was identical to that used in previous research on this topic (Rutchick et al., 2017). Participants used a machine (pictured in Supplemental Material) that ostensibly crushed ladybugs in what was described as a usability test. They were instructed to “kill as many insects as you’d like; make sure you kill at least two so that we have a good test.” Thus, participants could kill as few as two insects and up to the ten that were loaded into the machine on a conveyor belt device. In actuality, the insects were not killed; the “killing switch” operated a noisemaker.

### Post-study questionnaire

Once participants indicated that they were finished with the task, they completed a questionnaire. First, participants answered an open-ended question about their experience using the machine. Using 9-point Likert-type scales, participants then responded to three questions to reinforce the cover story (rating the machine’s difficulty, effectiveness, and comfort). Participants then indicated, on 9-point scales, their emotional reactions to the task, which constituted the measure of negative emotion: how enjoyable the ladybug-killing task was (reverse coded), how troubled they were by doing the task, and how upsetting it was to do the task. They then responded to several questions assessing potentially impactful features of remoteness. One item assessed abstract versus concrete construal of the task by asking participants how much they focused on the big picture (versus the details). One item assessed the degree to which participants felt that the benefits of using the machine outweighed the costs. Two items assessed the vividness of the experience: how real the task felt and how vivid (versus dull) it felt. Last, two items assessed subjective distance: how far they felt from the machine and how close they felt to the ladybugs (reverse coded).

After completing these measures, participants were probed for suspicion using a seven-part funnel debriefing procedure, fully debriefed, and dismissed.

### Manipulation of experimental conditions

Participants were randomly assigned to one of two conditions: Close (*n* = 107) and Remote (*n* = 108). Participants in the Close condition were in the same room as the machine, seated two feet from it. Participants in the Remote condition were in a different room, seated two feet from a computer screen, through which they viewed and heard the machine via videoconference software.

## Results

### Believability of the ruse

Funnel debriefing and examination of open-ended responses revealed that ten participants (seven Close and three Remote) did not believe that they were actually killing ladybugs, and were excluded from analyses. The final sample, then, consisted of 205 participants (100 in the Close condition and 105 in the Remote). The believability rate (95.3%) and ratings of the machine’s effectiveness in killing ladybugs (7.79/9) strongly suggest that the ruse was convincing.

### Analytic strategy

First, we examined the effects of remoteness on (ostensible) killing, negative emotion, and the candidate features. Next, a series of path models were tested using the Lavaan package^1^ in R for both simple (one mediator) and complex (serial mediators) indirect effects. Confidence intervals for the coefficients and the tests for indirect effect were tested using bootstrap-estimated SEs with 5000 samples (Shrout & Bolger, 2002). The simple mediation models tested both hypothesized (i.e., an indirect effect on killing via emotion, and an indirect effect on emotion via killing) and exploratory (i.e., construal, perceived benefit, vividness, and subjective distance) indirect effects.

### Effects of remoteness

An independent-samples t-test revealed that the difference in killing between the Close (*M* = 4.37 insects killed beyond the minimum) and Remote (*M* = 4.96) conditions was not statistically significant,* t*(203) = 1.31, *p* = 0.19, Cohen’s *d* = 0.18 (95% CI [− 0.09, 0.46]). A second t-test revealed that Remote participants experienced less negative emotion (a composite of “troubled” “upset,” and reversed “enjoyment”; *M* = 5.06) than Close participants (*M* = 5.91),* t*(203) = 3.00, *p* = .003, *d* = 0.42 (95% CI [0.14, 0.70]). In addition, Remote participants felt more subjectively distant (*M* = 5.12) from the insects and the machine than Close participants (*M* = 4.17), *t*(196) = 4.14, *p* < 0.001, *d* = 0.59 (95% CI [0.30, 0.87]). No other contextual features differed significantly by condition.

**Fig. 1:**
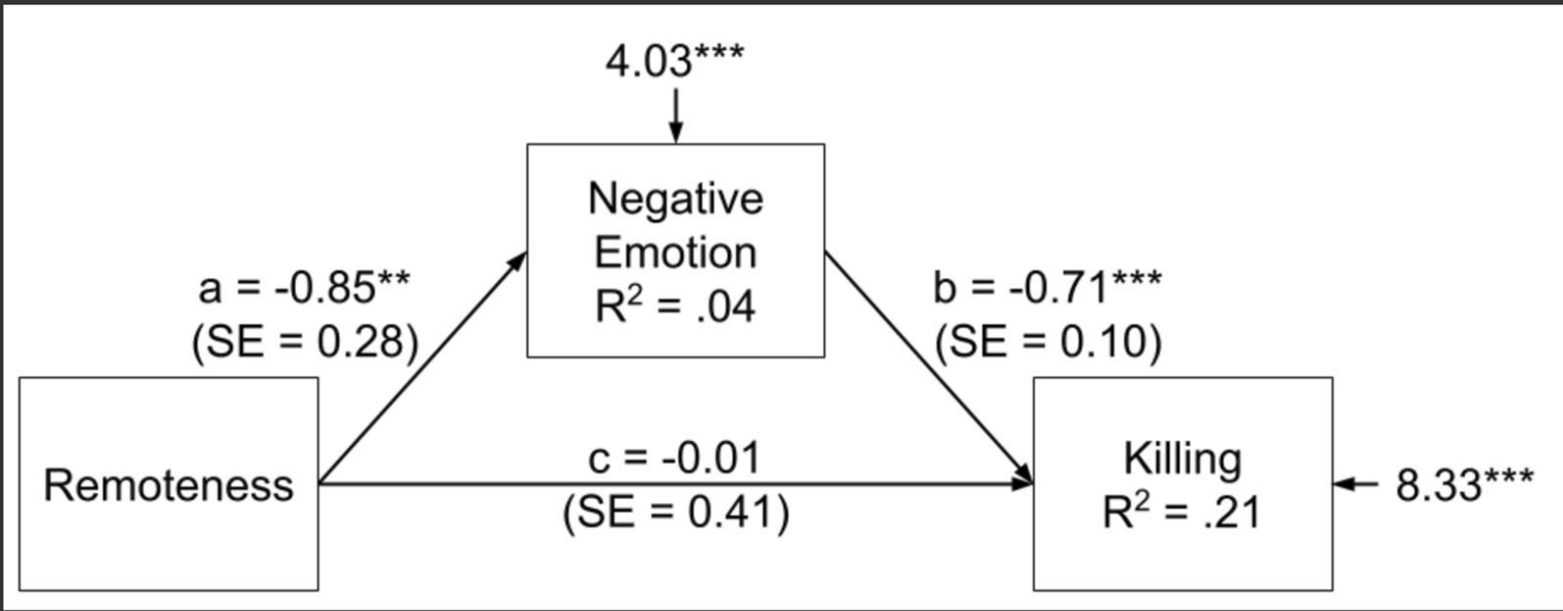
Indirect effect of remoteness on killing via negative emotion

Although remoteness did not directly influence killing behavior, it did have an indirect effect: Remote participants experienced less negative emotion (*b* = − 0.85, 95% CI [− 1.40, − 0.30], *SE* = 0.28, *Z* = − 3.02, *p* = 0.003, *β* = − 0.21), which led to killing more insects (*b* = − 0.71, 95% CI [− 0.91, − 0.52], *SE* = 0.10, Z = − 7.12, *p* < 0.001, *β* = − 0.45). The indirect coefficient was significant, *b*_sobel_ = 0.61 (95% CI [0.18, 1.03]), *SE* = 0.22, *Z* = 2.78, *p* = 0.005, *β* = 0.09 (Fig. 1).

### *Indirect effect of remoteness on killing *via* negative emotion.*

**Fig. 2:**
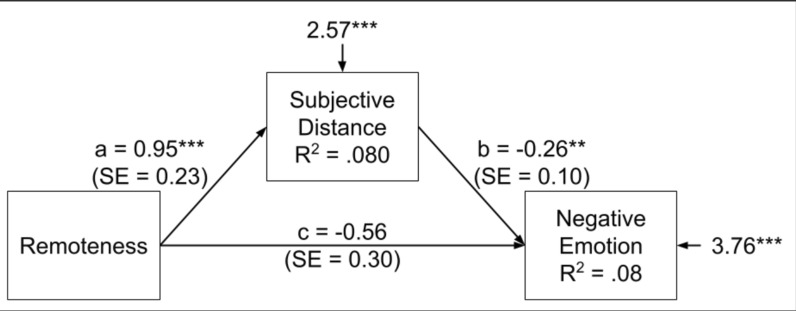
Indirect effect of remoteness on negative emotion via subjective distance

With respect to the effect of remoteness on negative emotion, there was an indirect effect, such that Remote participants perceived more distance (*b* = 0.95, 95% CI [0.50, 1.40], *SE* = 0.23, *Z* = 4.16, *p* < 0.001, *β* = 0.28), which led to less intense negative emotion (*b* = − 0.26, 95% CI [− 0.43, − 0.10], *SE* = 0.08, Z = − 3.07, *p* = 0.002, *β* = − 0.22). The direct prediction of negative emotion by condition was no longer significant, *b* = − 0.56, 95% CI [− 1.12, 0.01], *SE* = 0.29, *Z* = -1.93, *p* = 0.05, *β* = − 0.14. The indirect coefficient was significant*, b*_sobel_ = -0.25, 95% CI [− 0.45, − 0.05], *SE* = 0.10, *Z* = − 2.47, *p* = 0.01, *β* = − 0.06 (Fig. 2).

### *Indirect effect of remoteness on negative emotion *via* subjective distance.*

In addition, we examined models containing remoteness, emotion, killing, and the contextual features, which are presented in Supplemental Material.

## Discussion

In the current study, remoteness significantly decreased negative feelings about killing, but did not significantly increase killing behavior. This contrasts slightly with extant research using this paradigm (cf. Rutchick et al., 2017), which found a significant difference in killing behavior but not negative emotion. Effects in both studies were in the same direction, however, and an exploratory analysis on the combined data (presented in Supplemental Material) showed that, although there are some disparate patterns of significant pathways and indirect effects, the models do not differ significantly across the studies. The temporal sequence of these outcomes is unclear. One could experience negative emotion while anticipating killing, while killing, or when reflecting on having killed. In practice, particularly in an iterative task like this one, all three are likely true. The indirect effect described here suggests that negative emotion (as measured retrospectively), which is reduced by remoteness, may exert an inhibitory influence on killing.

A second goal of the current study was to consider how remoteness might induce these effects. We examined four possibilities: more abstract construal of the task, greater perceived benefit of the task, decreased vividness of the experience, and greater subjective distance from the target, all of which could plausibly increase killing behavior and decrease negative emotion. Here, subjective distance was the feature most strongly affected by the remoteness manipulation and was the only one that mediated a significant indirect effect. Indeed, the effects of remoteness on negative emotion and on killing behavior (via negative emotion) were both mediated by subjective distance. Relative to remote participants, participants in the close condition had access to a wider range of sensory cues—visual detail, sound, and tactile feedback from operating the machine—that may have heightened the salience of the experience and strengthened memory encoding. Sensory-rich events, particularly those involving negative affect, are typically associated with enhanced memory and intensified emotional responses (Kensinger, 2009; Laney et al., 2004). This suggests, consistent with Williams et al. (2014), that psychological distance (and, per Abraham et al., 2023, specifically psychological distance rather than abstract construals) reduces the intensity of experienced affect, and that this blunting of emotional responses is linked to more killing behavior.

These findings have implications for post-traumatic stress disorder and other consequences faced by many veterans. PTSD is associated with a range of both short-term and long-term negative emotions that is initiated by exposure to a significant trauma. Typically, one cannot be diagnosed with PTSD if they have witnessed traumatic events remotely – as in electronic media, television, movies, or pictures (American Psychiatric Association, 2022). This distance criterion tacitly supports our argument that psychological distance reduces the intensity of negative emotional states found in this study and in prior research substantiating the dose–response effect of exposure to trauma in military personnel later diagnosed with PTSD (Dohrenwend et al., 2006). Momentarily putting aside the diagnostic criteria excluding remote witnessing of trauma, remoteness in the current study did decrease negative feelings about killing, which could serve as a buffering mechanism for post-traumatic psychiatric concerns. Remote participants may have reappraised the killing task by framing it in less aversive terms, supported by greater cognitive control over their emotions (Ochsner & Gross, 2005). Relatedly, people sometimes cope with overwhelming situations by mentally detaching from the experience as a defensive strategy (Marmar et al., 1994; Van Der Kolk, 1997). While the subjects in the present study clearly did not experience the same kind or degree of trauma that military personnel face, the mechanism by which the trauma manifests in negative affect may be similar. Although it is unclear whether participants in the present study engaged in dissociative thinking (common among military personnel in peritraumatic conditions; cf. Gibney et al., 2013), this would be an interesting avenue of future research.

Generalizations from the current findings should be made cautiously, however. Although the university from which the sample was drawn is diverse, its students are younger (and may therefore interact differently with technology) than the general or military population. They were also located in the United States, and attitudes toward killing (and indeed toward insects) might well be different in other parts of the world. More broadly, the killing task was carried out on insects, in a laboratory, in the context of a usability test. Although participant responses strongly suggest that the experience felt psychologically real, this does not imply an equivalent psychological process to the act of killing in a military (or any non-laboratory) context.

Future research could consider other factors that influence the moral weight of killing. Work on moral injury (Shay, 2014) argues that trauma often stems less from killing itself than from judgments of its necessity and justification. It may be, for example, that a sniper or remote operator may come to view an enemy differently after prolonged surveillance, becoming aware of their social connections and everyday routines. Indeed, recent research (Norrholm et al., 2023) has documented stressors specific to remote operators. How judgments of necessity and moral evaluations of the target might interact with remoteness is not yet known, and examining this question would expand the applicability of this research into both military and civilian contexts.

Beyond the context of killing and PTSD, the technological conditions by which remoteness was created are relevant to many military and civilian workplace environments. Communicating via videoconference is more remote than communicating in person, and email is more remote than a handwritten message (Kiesler et al., 1984; Walther, 1996). It is possible that remoteness reduces inhibitions about aggressiveness in tone and diction. For example, in a face-to-face interaction, the target’s reactions (such as facial expressions and other nonverbal behavior) are directly witnessed by the aggressor. However, in technologically mediated communication, the aggressor may not directly witness the victim’s reaction. This can increase disinhibition, impulsivity, and the intensity of aggression (Culnan & Markus, 1987; Postmes et al., 1998; Teich et al., 1999; Wright & Li, 2013; although see Walther, 1993 and Walther & Burgoon, 1992). One need only examine the comments under an online article to see vitriol and profanity that clearly surpasses what is typical in face-to-face conversation (Seely, 2018).

The current findings reinforce the potential role of technology in creating distance, and demonstrate the affective and behavioral impact of that distance. Undoubtedly, remoteness-inducing technology can afford material benefits, such as the use of drones to decrease the number of soldiers on the ground. Concurrently, as this research shows, the distance induced by remoteness can reduce the negative emotion surrounding killing. This blunting implicates the initiation of killing behavior and the immediate emotional impact of killing, and may also potentially buffer post-traumatic stress. In both combat and other military settings, then, it is crucial to understand the influence of technological mediation and the distance it affords.

## Supplementary information

Additional file 1.

## Data Availability

The datasets generated and analyzed during the current study are available in the OSF repository at https://osf.io/hm9yj/.

## Footnotes

1 The number of kills was distributed non-normally. When analyses were conducted adjusting for non-normality (robust estimates) or treating kills as count data, conclusions did not differ appreciably. We present analyses with assumptions of normality for simplicity of interpretation.

## Declarations

### Open practices statement

Data and analysis scripts are available at https://osf.io/hm9yj/. The experiment was not preregistered (it was conducted in 2016, before preregistration became widely adopted).
