# Supplementary material for: Remoteness decreases negative feelings about killing
Source: Cogn Res Princ Implic. 2026 Feb 24;11:16. doi: 10.1186/s41235-026-00706-0 (PMC12932782; doi:10.1186/s41235-026-00706-0)
Supplement: Supplementary file 1 — Additional file 1. [file 41235_2026_706_MOESM1_ESM.docx]

Supplemental Material

*Images of Ostensible Insect-Killing Machine and Controller*


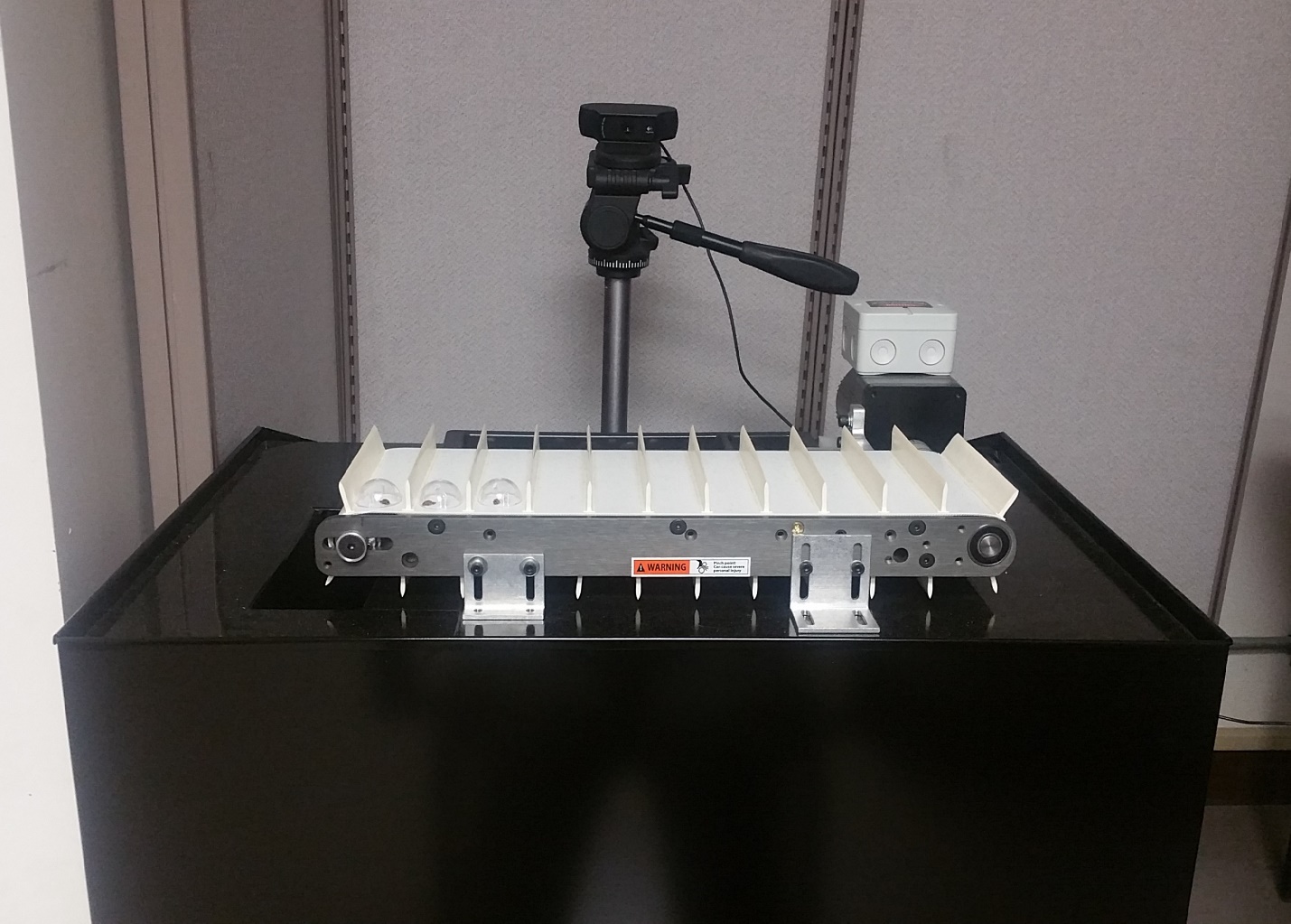
Figure S1. Top of insect-killing machine. Note plastic hemispheres containing live ladybugs on conveyor belt, with hole in top of machine leading to killing chamber at left of conveyor belt.

*
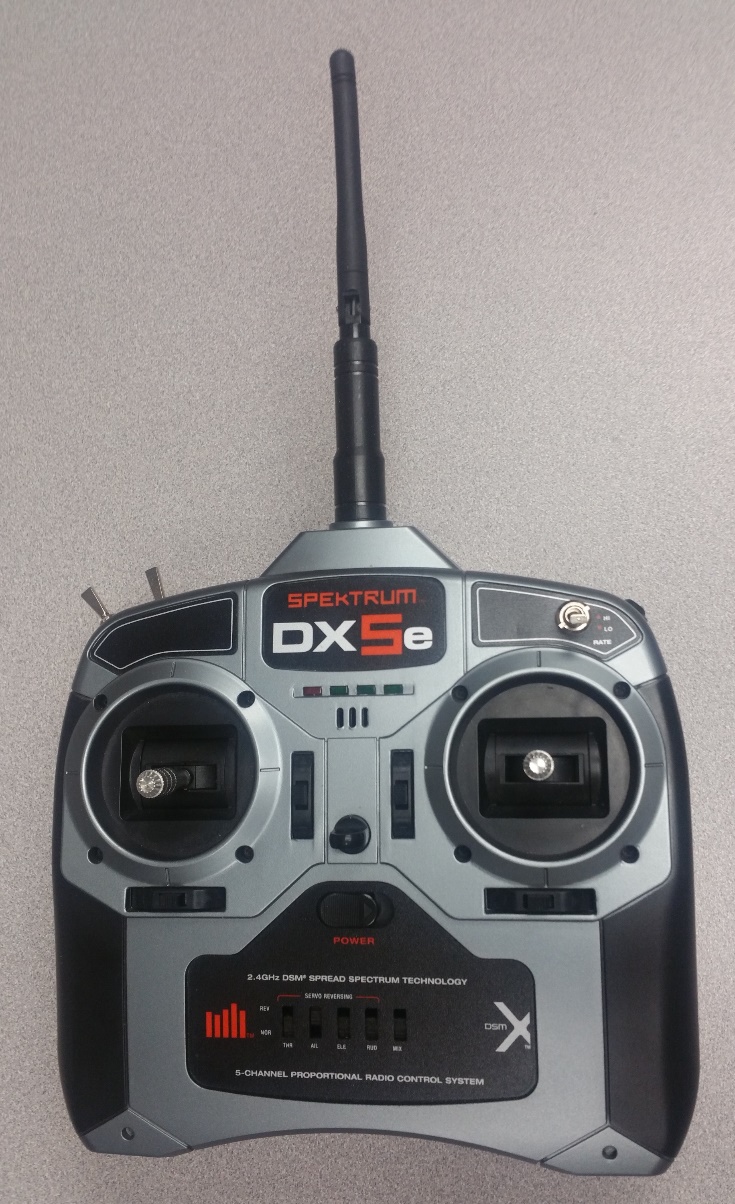
*

Figure S2. Remote used to control insect-killing machine. The joystick on the right, if pushed to the left, advances the conveyor belt, dropping a ladybug-containing capsule into the killing chamber. The joystick on the left, if pushed up, operates the killing chamber (actually a computer fan spinning against a nail that produces a grinding sound).

*Post-task Questionnaire Administered to Participants*

1. (Open-Ended)
   Please describe your experience using the machine. Discuss anything you’d like – this could include anything at all about what it was like to complete the insect-killing task using the machine. Please take a minute or so to write a thorough response. (Your response will be confidential.)
2. How difficult was the machine to operate? (1 = Very Easy; 9 = Very Difficult)
3. How effective was the machine in grinding up the ladybugs? (1 = Not At All Effective; 9 = Very Effective)
4. How comfortable did you feel when you were using the machine? (1 = Not At All Comfortable; 9 = Very Comfortable)
5. How enjoyable was the ladybug-killing task? (1 = Not At All Enjoyable; 9 = Very Enjoyable)
6. How troubled were you by doing the ladybug-killing task? (1 = Not At All Troubled; 9 = Very Troubled)
7. How upsetting was it to do the ladybug-killing task? (1 = Not At All Upsetting; 9 = Very Upsetting)
8. How far away did you feel from the machine? (1 = Not Far Away At All; 9 = Very Far Away)
9. How close did you feel to the ladybugs? (1 = Not Close At All; 9 = Very Close)
10. Some experiences feel very unreal (like they are not actually happening to you), whereas other experiences feel very real (like they are actually happening to you). The ladybug-killing task felt: (1 = Very Unreal; 9 = Very Real)
11. Some experiences feel very dull, whereas other experiences feel very vivid. The ladybug-killing task felt: (1 = Very Dull; 9 = Very Vivid)
12. When we think about our experiences, sometimes we focus more on the big picture, whereas other times, we focus more on details and procedure. During the ladybug-killing task, I focused more on: (1 = The Details; 9 = The Big Picture)
13. Sometimes costs outweigh the potential benefits of an action. Sometimes potential benefits outweigh the costs of an action. As mentioned before the task, the use of these machines requires killing ladybugs for the benefit of collecting biological samples for scientific research, and for extracting dye for coloring of clothing. In this case, I think that: (1 = The cost [killing ladybugs] is definitely NOT worth the potential benefits; 9 = The cost [killing ladybugs] is definitely worth the potential benefits)

*Funnel Debriefing Questions (administered orally)*

1. Do you have any other thoughts or reactions about this study?
2. Was anything confusing about the study?
3. What did you think the main purpose of the study was?

3a. (Distant Condition) Do you recall where the insect-killing machine is located?

1. Was there anything strange about the machine?
2. Was anything suspicious about the study?
3. Do you think the machine was killing the ladybugs?
4. The machine was NOT actually killing the ladybugs. Did you think it was at any time throughout the study?

*Correlations and Reliabilities of Multi-item Composites*

Participants indicated, on 9-point scales, their emotional reactions to the task, which constituted the measure of negative emotion (α = 0.82): how enjoyable the ladybug-killing task was (reverse coded), how troubled they were by doing the task, and how upsetting it was to do the task.

Two items assessed the vividness of the experience (*r* = .47, *p* < .001, *n* = 201): how real the task felt and how vivid (versus dull) it felt. Last, two items assessed subjective distance (*r* = .21, *p* < .001, *n* = 198): how far they felt from the machine and how close they felt to the ladybugs (reverse coded).

**Table S1.** *Effects of Remoteness*

|  | Remote | Close |
| --- | --- | --- |
|  | Mean (SD) | Mean (SD) |
| Number of Kills | 4.96 (3.23) | 4.37 (3.24) |
| Negative Emotion**** | 5.06 (2.05) | 5.91 (1.98) |
| Subjective Distance** | 5.12 (1.75) | 4.17 (1.45) |
| Vividness | 5.56 (1.90) | 5.51 (1.94) |
| Construal Level | 4.91 (2.37) | 5.13 (2.75) |
| Benefit of Machine | 5.14 (2.13) | 4.64 (2.38) |

*Note.* Descriptive statistics for all direct effects. Dotted line distinguishes between hypothesized outcomes of remoteness and candidate mechanisms. **p < .01.

*Complex Indirect Effects of Remoteness on Killing Behavior and Negative Emotion*

Last, models containing remoteness, emotion, killing, and the various candidate mechanism variables were examined (see Figure S3 for conceptual framework). Only one model yielded a significant serial mediated indirect effect (see Figure S4 for unstandardized values and significant pathways).


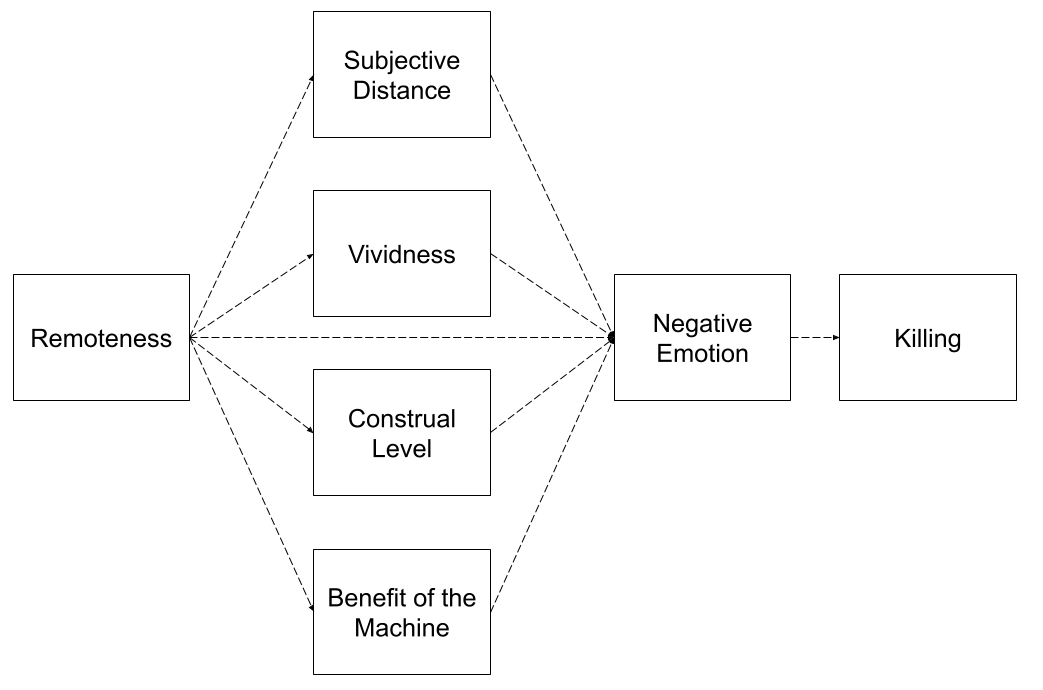


Figure S3. Conceptual framework showing (non-concurrent) mediational analyses examining candidate mechanisms.

*Direct paths.* Participants in the Remote condition felt greater subjective distance (a composite of reversed closeness to the insects and distance from the machine) (*b* = 0.95, *SE* = 0.23, *Z* = 4.16, *p* < 0.001, 95% CI for *b* [0.50, 1.40]) which in turn led to feeling less intense negative emotion (*b* = -0.26, 95% CI [-0.43, -0.10], *SE* = 0.09, *Z* = -3.07, *p* = 0.002, *β* = 0.03), but perceiving greater distance did not lead directly to significantly more killing behavior (*b* = 0.05, 95% CI [0.20, 0.31], *SE* = 0.13, *Z* = 0.39, *p* = 0.70_,_ *β* = 0.03). Furthermore, participants in the Remote condition no longer felt significantly less intense negative emotion (*b* = -0.56, 95% CI [-1.12, 0.01], *SE* = 0.29, *Z* = -1.93, *p* = 0.05, *β* = 0.14), but feeling less intense negative emotion did lead to more killing behavior (*b* = -0.71, 95% CI [-0.92, -0.51], *SE* = 0.11, *Z* = -6.80, p < .001, *β* = -0.45). Condition did not directly predict killing behavior within the model, *b* = 0.13, *SE* = 0.43, *Z* = 0.30, *p* = 0.76, 95% CI for *b* [-0.71, 0.97].

*Indirect paths*. The Remote condition led to feeling less intense negative emotions via participants’ perceptions of distance, *b*_sobel_ = -0.25, 95% CI [-0.45, -0.05], *SE* = 0.10, *Z* = -2.47, *p* = 0.01, *β* = -0.06. Additionally, participants’ perceptions of distance predicted an increase in killing behavior via reported negative emotion, *b*_sobel_ = 0.19, 95% CI for *b_sobel_* [0.06, 0.32], *SE* = 0.07, *Z* = 2.80, *p* < 0.01, *β* = 0.10. Finally, participants in the Remote condition showed an increase in killing behavior via both distance and negative emotion, *b_sobel_* = 0.18, 95% CI [0.03, 0.33], *SE* = 0.08, *Z* = 2.32, *p* = 0.02, *β* = 0.03. All other indirect paths were not significant. These analyses suggest, then, that remoteness increased subjective distance and attenuated negative emotion, and in this way indirectly increased killing behavior.

*
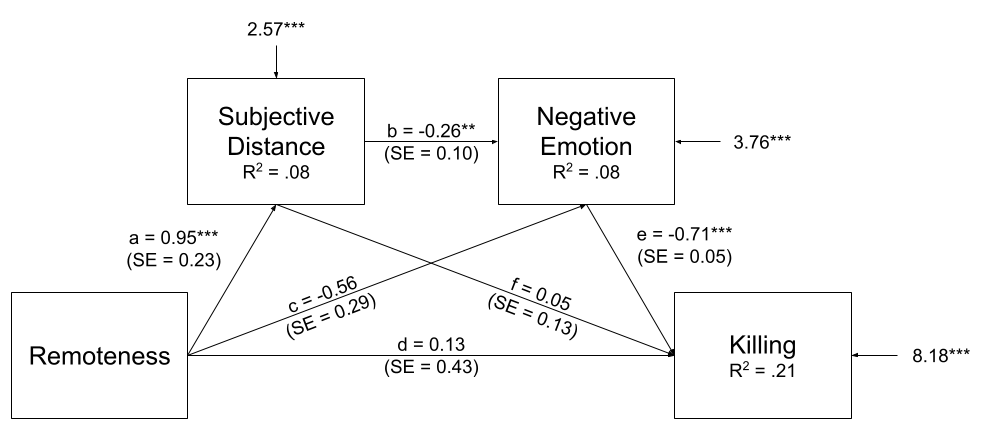
*

Figure S4. Path model showing serial indirect effect of remoteness on killing.

*Effects of Gender*

There was a main effect of gender on negative emotion: females (*M* = 5.69) reported experiencing significantly more negative emotion than males (*M* = 4.89; *t*(203) = 2.49, *p* = 0.01, Cohen’s *d* = 0.39, 95% CI [0.08, 0.70]. There were no other significant main effects of gender, and it did not interact with remoteness to influence any dependent variable.

*Analyses of the Direct and Indirect Effects of Remoteness Across Studies*

As the current study extends extant research using this paradigm (Rutchick et al., 2017), we also examined mediation models across both studies in an exploratory approach. Thus, a multi-group analysis comprising the current data (*N* = 205) and the data from Rutchick et al., 2017 (*N* = 317) was conducted in order to examine the aggregate effects of remoteness, which differed somewhat between the two studies. Using fully constrained (i.e., equality constraints on the regression coefficients, predictor variances and error variances across the two data sets) multi-group analyses, we tested both the indirect effect of remoteness on killing behavior through negative emotions and the indirect effect of remoteness on negative emotion through killing behavior in order to both compare the results of the two data sets and to establish if one mediation model is a better fit.

*Direct and indirect effects on killing behavior.* The multi-group model predicting killing behavior indicates that the data from the two groups are equivalent, as the fully constrained model fit the combined data well, *χ*^2^(6) = 10.32, *p* = 0.11, CFI = 0.95, RMSEA = 0.05. The direct path from remoteness to killing behavior was significant (*b* = 0.62, *SE* = 0.27, *Z* = 2.29, *p* = 0.02, 95% CI for *b* [0.09, 1.15], *β* = 0.09), as was the direct path from remoteness to negative emotion (*b* = -0.47, *SE* = 0.18, *Z* = -2.53, *p* = 0.01, 95% CI for *b* [-0.83, -0.11], *β* = -0.11). The path from negative emotion to killing was significant (*b* = -0.56, *SE* = 0.06, *Z* = -8.74, *p* < 0.001, 95% CI for *b* [-0.69, -0.44], *β* = 0.09) and so was the indirect effect from remoteness to killing behavior via negative emotion (*b*_sobel_ = 0.26, *SE* = 0.11, Z = 2.43, *p* = 0.02, 95% CI for *b_sobel_* [0.05, 0.47], *β* = 0.04).

*Direct and indirect effects on negative emotion.* The multi-group model predicting negative emotion also indicates that the data from the two groups are equivalent as the fully constrained model fit the combined data well, *χ*^2^(6) = 10.32, *p* = 0.11, CFI = 0.95, RMSEA = 0.05. The direct path from remoteness to negative emotion was not significant (*b* = -0.27, *SE* = 0.17, *Z* = -1.53, *p* = 0.13, 95% CI for *b* [-0.60, 0.08], *β* = 0.06), however the direct path from remoteness to killing was significant (*b* = 0.88, *SE* = 0.29, *Z* = 3.06, *p* = 0.002, 95% CI for *b* [0.32, 1.45], *β* = 0.13). The path from killing to negative emotion was significant (*b* = -0.23, *SE* = 0.03, *Z* = -8.74, *p* < 0.001, 95% CI for *b* [-0.28, -0.18], *β* = 0.36) and so was the indirect effect from remoteness to negative emotion via killing behavior (*b*_sobel_ = -0.20, *SE* = 0.07, *p* = 0.004, 95% CI for *b* [-0.34, -0.07], *β* = -0.05).
